# First International Workshop on Zika Virus Held by Oswaldo Cruz Foundation FIOCRUZ in Northeast Brazil March 2016 – A Meeting Report

**DOI:** 10.1371/journal.pntd.0004760

**Published:** 2016-06-03

**Authors:** Rafael F. O. Franca, Maria Helena L. Neves, Constancia F. Junqueira Ayres, Osvaldo P. Melo-Neto, Sinval P. Brandão Filho

**Affiliations:** Instituto Aggeu Magalhães (IAM), Fundação Oswaldo Cruz (Fiocruz), Recife, Pernambuco, Brazil; Pediatric Dengue Vaccine Initiative, UNITED STATES

In the first two days of March 2016, an International Workshop on Zika Virus was held in the city of Recife, Brazil, hosted by the Oswaldo Cruz Foundation (Fiocruz). The workshop took place at the Research Institute Aggeu Magalhaes (IAM), the state of Pernambuco research branch of Fiocruz, responsible for hosting the meeting and its organization. Representatives from several institutions working on Zika virus science attended, including researchers and academics from several other Fiocruz research entities (based in Rio de Janeiro, Belo Horizonte and Bahia), from major Brazilian Universities (University of Sao Paulo, Federal University of Rio de Janeiro and others) and from other Brazilian research centers (such as the Evandro Chagas Institute—IEC). Foreign participants included representatives from the National Institute of Allergy and Infectious Diseases NIAID-USA and the University of Glasgow, among others. The Workshop brought together more than 600 participants and was accessible, live, through the web (over four thousand accessions from 26 countries). The purpose of the meeting was to provide an updated view on Zika virus in Brazil and to discuss recent advances and challenges on its research. With the recent Zika virus outbreak in Northeast Brazil and especially the remarkable increase in newborns suffering a congenital disorder termed microcephaly—a rare neurological condition in which an infant's head is significantly smaller than the heads of other children of the same age—recently linked to Zika virus infection during pregnancy [1–5], the Brazilian public health authorities declared a National Public Health Emergency on Nov 11, 2015. This was followed by the recognition by the World Health Organization—WHO, on February 1, 2016, of the cluster of microcephaly cases and other neurological disorders as a health emergency, declaring a Global Emergency and the spread of the Zika virus an “extraordinary event” with a public health threat to other parts of the world (source: http://www.who.int/emergencies/zika-virus/en/).

During the meeting, special sessions were organized by experts in the following research fields: ***A******rboviruses*, *Zika and vaccines***–discussed data regarding different circulating arthropod borne viruses in Brazil (such as chikungunya and dengue), new data about Zika virus biology, *in vitro* models of infection and also strategies for vaccine development; ***B******iology of the virus vector interaction***–focused on new findings on virus vector studies, including an evaluation of potential vectors for Zika virus and countermeasures to mosquito control; **C****linical**–with the intention of discussing the last clinical findings on Zika virus infection and major birth congenital malformations; ***D******iagnosis***—plotted new strategies to improve laboratory tests, development of technological products and also a historical update of Zika virus identification in Brazil; ***E******pidemiology***—this session was designed to provide an update on the clinical epidemiological studies under development in Brazil, with a presentation of the data on a case-control investigation of microcephaly cases and cohort studies.

**Fig 1:**
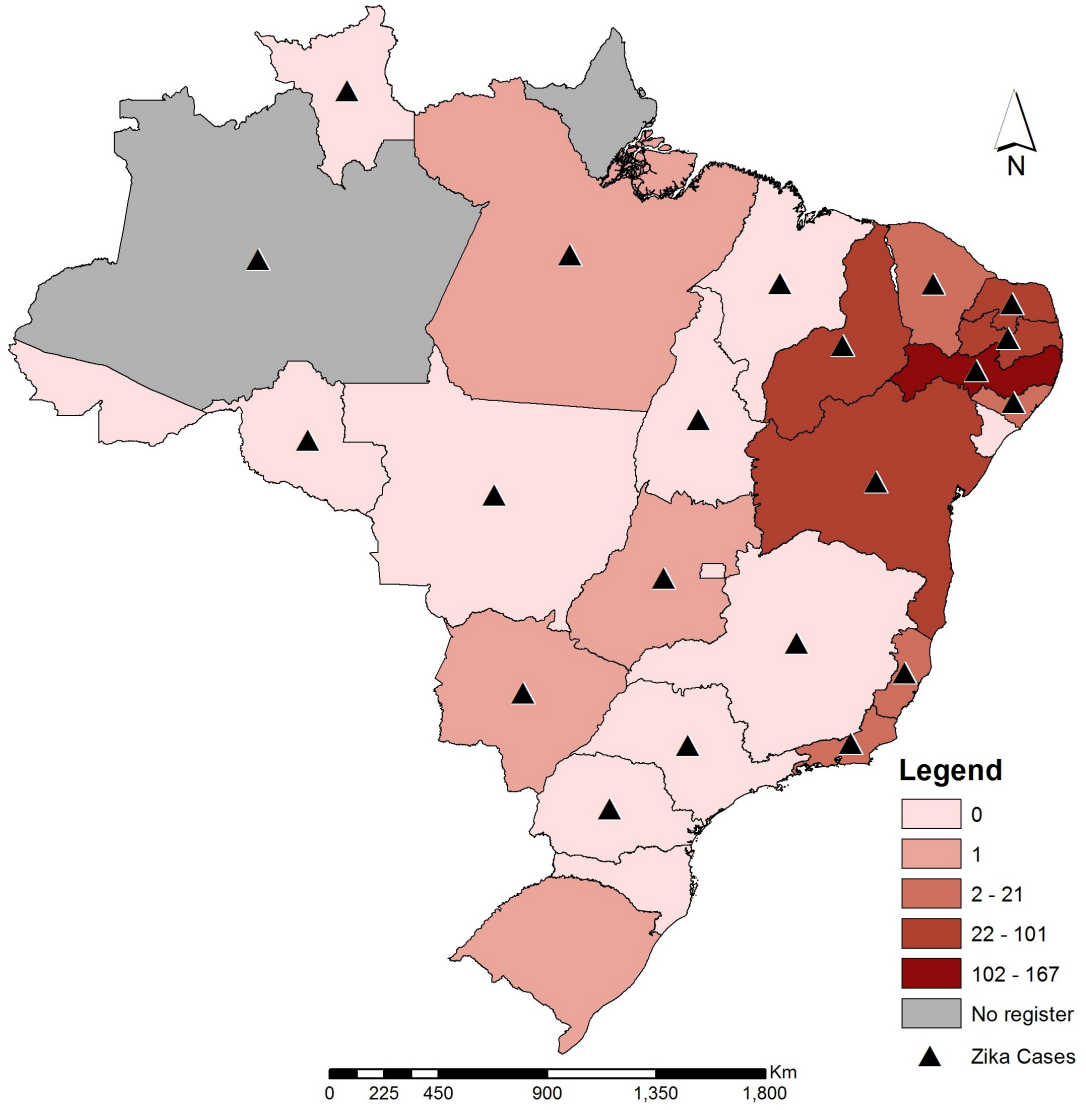
Brazilian federative units with Zika documented autochthonous cases (triangles) and number of microcephaly confirmed cases in each federal state (color shading of the affected Brazilian states) (source: Brazilian Ministry of Health and Secretary of Vigilance in Health).

The first day the meeting opened with a talk from the IEC team on Zika virus infection and the global challenge to control its spread. Subsequent talks covered the clinical manifestations presented by Zika infected individuals, from a medical perspective. Medical doctors from Northeast Brazil reported that the major clinical findings on microcephaly confirmed cases were calcifications (approximately 98%), ventriculomegalia and disorder of cortical development, including simplification of gyrus and lissencephaly with a lower proportion of other abnormalities such as pachygyria. Official data from the Brazilian Ministry of Health confirmed that currently Zika virus is circulating in 22 of the 27 states in Brazil, with two confirmed deaths (Fig 1). By March 1, a total of 5.909 cases of microcephaly were reported and are currently under investigation. From 641 confirmed microcephaly cases, the number of newborn deaths reached 139 (stillbirths and/or newborn deaths) and from the total number of microcephaly cases investigated, 82 were confirmed to be Zika by laboratory tests (RT-qPCR and/or serology). Most of the cases were reported from the state of Pernambuco (1.672 cases) (source: Brazilian Ministry of Health).

On the Diagnosis panel, the discussions were mainly on the development of new laboratory tools. Given the extensive cross reactivity with other circulating arboviruses in Brazil, the lack of reliable and specific serologic tests for Zika virus represents a major challenge. Currently, only two serological tests are licensed for Zika virus diagnosis in Brazil (Anti-Zika Virus ELISA IgG/IgM and IIFT Arboviral Fever Mosaic 2 IgG/IgM, both from EUROIMMUN). In addition to serological tests, Fiocruz is providing a recently licensed multiplex Dengue/Zika and Chikungunya assay based on NAT technology (nucleic acid test or nucleic acid amplification test). Discussions about development and characterization of monoclonal antibodies and recombinant proteins, as biotechnological products to be applied on different platforms for Zika virus diagnostic, occurred during the event, however no new kits are being licensed at the moment, which could be explained, in part, by the lack of reliable serum panels for kits validation. Researchers from IAM investigating the microcephaly cases reported the detection of Zika virus specific IgM in roughly 90% of the samples of cerebrospinal fluid (CSF) tested from microcephalic newborns, which strongly correlates Zika virus infection with brain malformation. The team from the Oswaldo Cruz Institute (IOC—a Fiocruz branch from Rio de Janeiro) provided results on Zika virus identification from several body fluids such as urine, semen (up to 2 months after symptoms), saliva and CSF. The data showed that Zika virus found in unusual samples (urine, saliva, semen) can persist for longer time periods than in the blood. So far, however, only sexual, perinatal and vector transmissions have been documented for Zika virus. Therefore, is concerning the fact that Zika virus detection on semen for long periods after the initial infection has serious implications on sexual transmission. Although organ transplantation and blood transfusion are probable transmission routes, there are no reports yet in the literature [6–11]. Despite the fact that new Zika candidates vaccines may not enter clinical trials until the next 2 or 3 years, scientific efforts to achieve a safe and efficient Zika vaccine are crucial to counteract this new emerged viral disease. Vaccine development is still very preliminary and to the moment only pre clinical studies are in course. On vaccine discussion topic, scientists presented different strategies for vaccine development, based on several immunization platforms, such as viral chimeras using the vaccinal Yellow Fever 17D strain, virus attenuation by reverse genetics (infectious clones) and subunit vaccines (DNA and/or recombinant proteins).

Mosquitoes from the genus *Aedes (Ae*. *albopictus* and *Ae*. *Aegypti)* are the main species responsible for Zika virus transmission in Brazil [12,13]. During the Biology of the Virus Vector Interaction session, researchers from IAM reviewed the evidence regarding Zika virus transmission and presented results derived from laboratory experiments that implicate other mosquito species as potential vectors involved in the Zika virus life cycle. Using mosquitos artificially fed with Zika virus infected blood, they were able to detect the virus in the salivary glands of *Culex quinquefasciatus* 7 and 15 days post-feeding, confirming a high infection rate of 100% and 67%, respectively. In addition, advances and new approaches for mosquito control were presented in the session and challenging aspects for the implementation of control measures discussed, taking into account the socio-economical and environmental conditions of urban areas in Brazil.

Since its introduction in Brazil, Zika virus infection has spread rapidly through the tropical Americas and we hypothesize that sequential flaviviruses exposures can represent a complicating factor for its spread, especially in Brazil where rates for prior dengue infection are extremely high. Currently, a number of independent groups are developing projects on Zika virus investigation in Brazil. Recently, Fiocruz (the largest, health related, research institute in Latin America, with a total of 18 units scattered throughout different regions in Brazil) established a National Network Zika Committee through the integration of efforts carried out by several of its research branches. These efforts are focusing on the development of better diagnostic tools, vaccine research, virus genetic studies, immunopathologic studies on Zika patients and research on vector populations and control. Currently, Zika virus is still expanding within the Brazilian territory and in other countries from Central and South America, probably due to the availability/abundance of vectors and the challenges in achieving efficient levels of vector reduction with a consequent impact on virus transmission. It is currently very difficult to determine how and to where will the virus spread over time. Assumptions by field specialists testify that when herd immunity reaches a level of ~80%, Zika virus transmission could then drop, however this is only speculative and based on previous data from recent outbreaks of dengue and chikungunya [14]. Serious funding and science investments are thus critical in order to face this newly emerged disease.
